# Evaluation of Seismic Performance and Effectiveness of Multiple Slim-Type Damper System for Seismic Response Control of Building Structures

**DOI:** 10.1155/2014/189106

**Published:** 2014-09-11

**Authors:** David Kim, Eun Hee Sung, Kwan-Soon Park, Jaegyun Park

**Affiliations:** ^1^Department of Architectural Engineering, Dongguk University, Seoul 100-715, Republic of Korea; ^2^Department of Civil and Environmental Engineering, Dankook University, Yongin 448-701, Republic of Korea

## Abstract

This paper presents the evaluation of seismic performance and cost-effectiveness of a multiple slim-type damper system developed for the vibration control of earthquake excited buildings. The multiple slim-type damper (MSD) that consists of several small slim-type dampers and linkage units can control damping capacity easily by changing the number of small dampers. To evaluate the performance of the MSD, dynamic loading tests are performed with three slim-type dampers manufactured at a real scale. Numerical simulations are also carried out by nonlinear time history analysis with a ten-story earthquake excited building structure. The seismic performance and cost-effectiveness of the MSD system are investigated according to the various installation configurations of the MSD system. From the results of numerical simulation and cost-effectiveness evaluation, it is shown that combinations of the MSD systems can effectively improve the seismic performance of earthquake excited building structures.

## 1. Introduction

Strong earthquakes have occurred in many countries and have caused many problems including casualties, economic loss, destruction of infrastructures, and even leaking of radioactive materials. Therefore, earthquake resistant design and reinforcement of existing structures have become increasingly more important due to increasing probability of strong earthquakes. Especially, a higher level of earthquake resistance is required for infrastructures and industrial facilities as it has been shown in historical earthquake studies [1–3].

Several seismic resistance techniques are available, depending on the various types and conditions of structures. To enhance the stiffness and strength of structures, we can add a moment frame, shear wall, or bracing. However, the stiffness or strength enhancement causes plastic hinges at both ends of a column during strong earthquakes and it may finally yield a fragile failure [4–6]. Another method involves increasing the ductility of structural members by adding steel plates, glass fibers, and carbon fibers. While the method of ductility enhancement can prevent fragile failures, it is difficult to rehabilitate plastic hinges developed during earthquake movements [7, 8]. On the contrary, structures with energy dissipating devices can be easily repaired because damages are mainly concentrated and localized on the device during earthquakes. Thus, in order to improve the structural seismic performance and reduce the risk of damage or failure of existing structures, energy dissipation devices have been studied by many researchers and extensively used in seismic retrofitting techniques [9–16]. In particular, various viscous dampers can effectively control seismic responses due to earthquakes which have wide range of frequency contents and magnitudes. Viscous dampers generally dissipate energy through movements of a piston in a highly viscous fluid and a large number of viscous dampers have been developed and installed in structures [17–21]. They are installed between stories as diagonal braces in general and have many advantages such as stable performance, easy installation and replacement, applicability on both buildings in construction and buildings which are currently used, and reliability in energy dissipation. However, the deformation of the viscous damper is proportional to the relative displacement of the two neighboring stories such that a small relative displacement can limit the performance of the viscous damper. Therefore, larger capacity dampers are required to obtain desirable performances. As a result, the size of the damper is inevitably increased and a larger installation space is thus required, which may decrease the space availability of buildings. Sometimes, this can further restrict a resident's view and movement [22].

A new multiple slim-type damper system has recently been developed [23] not only to achieve reliable seismic performance by the installation of damping devices but also to solve the problem of structural space availability. The main objective of this study is to validate the feasibility and effectiveness of the developed MSD system in terms of practical implementation and economy.

This study investigates the seismic performance of the proposed MSD system. Dynamic loading tests are carried out with three MSD systems manufactured at a real scale and a nonlinear time history analysis for a ten-story earthquake excited building structure is performed. The cost-effectiveness of the MSD system is also investigated according to the various installation configurations of the MSD system and comparative results are presented.

## 2. Multiple Slim-Type Damper

A multiple slim-type damper (MSD) was developed to improve the adaptability of conventional dampers [23]. In this section, the development and results of dynamic loading tests of the MSD system in [23] are briefly summarized to introduce the mechanical model of the MSD system, which will be used for numerical simulations in this study.

### 2.1. Development

**Figure 1:**
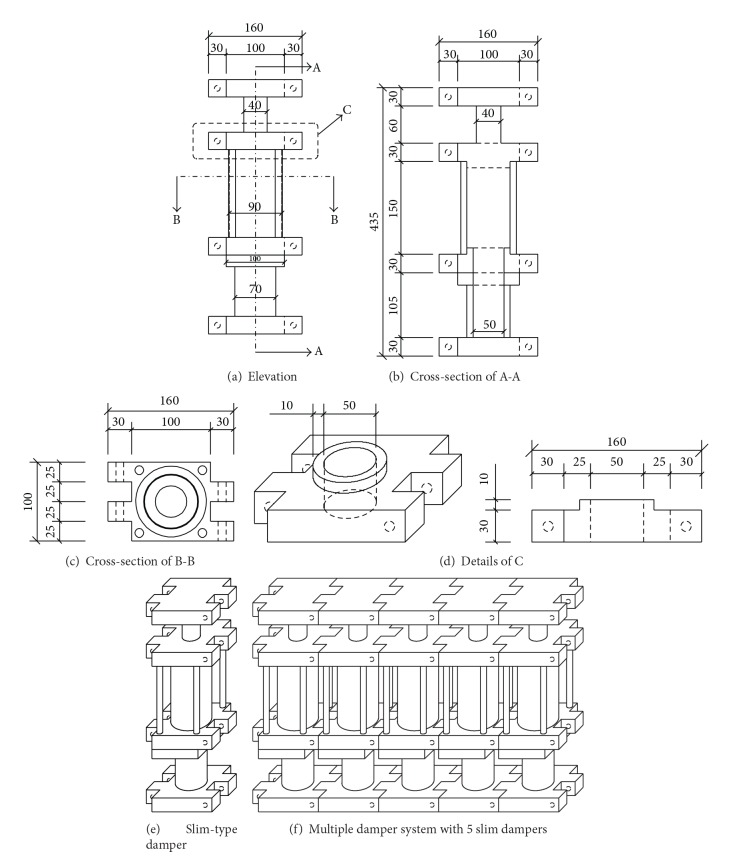
Drawings of multiple slim-type dampers.

Drawings of the developed MSDs are presented in Figure 1. An MSD is composed of several slim-type dampers which have smaller diameters than those of conventional dampers. Unlike conventional dampers, slim dampers are arranged in parallel. In general, the MSD can be installed in the wall using diagonal bracing. Combinations of slim dampers make it possible to control seismic performance properly with smaller installation spaces. As a result, the thickness of the wall is not necessarily increased to install seismic dampers that are typically equipped in the wall.

**Figure 2:**
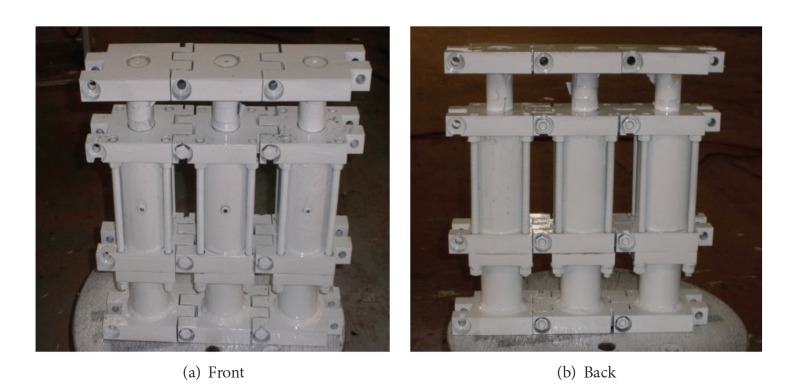
Assembled TSD with three slim-type dampers.

In order to investigate the performance of the MSD, several slim dampers are manufactured and two MSDs are assembled. The MSD with three slim dampers is named the tri-slim-damper (TSD) and the MSD with five slim dampers is named the penta-slim-damper (PSD). Also, for convenience the MSD with one slim damper is named the single-slim-damper (SSD). As an example, Figure 2 shows the assembled TSD.

### 2.2. Loading Test

**Figure 3:**
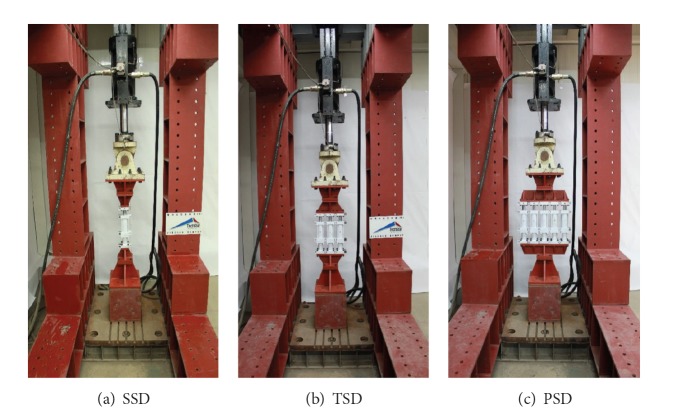
Dynamic loading test.

A dynamic loading test is performed at Ajou University in Republic of Korea in order to determine the dynamic characteristics and seismic performances of the three different developed systems, that is, SSD, TSD, and PSD. In the cyclic loading test, the loading velocity of the actuator is selected as the controlled variable and the loading velocities of 2 mm/sec, 3 mm/sec, 4 mm/sec, 5 mm/sec, 7 mm/sec, and 10 mm/sec are used. Figures 3(a), 3(b), and 3(c) show dynamic test setups of SSD, TSD, and PSD, respectively.

**Figure 4:**
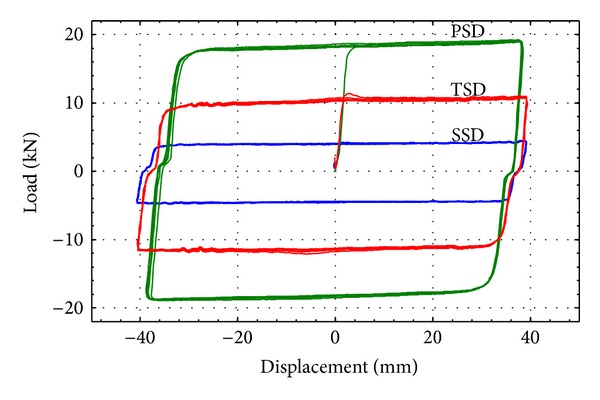
Load-displacement hysteresis curve (loading velocity = 5 mm/sec).

As a result of the test, load-displacement hysteresis curves are obtained for all loading cases. For a loading velocity of 5 mm/sec, the hysteresis curve is depicted in Figure 4. As the loading and unloading proceed, hysteresis loops are well developed. Also, the linear and plastic behaviors are clearly observed in Figure 4. During the unloading process, the displacement is decreased in parallel with the initial portion of the loading curve. It is also natural that the maximum damping forces in the loop are increased as the number of slim-dampers used is increased.

The manufactured MSDs show general curve shapes similar to those of conventional viscous dampers. Therefore, it is found that the developed MSDs can be used as damping devices for the vibration control of earthquake excited structures.

In addition, maximum damping forces are summarized in Table 1.

As the velocity increases, the maximum damping force increases gradually. However, the linear relationship between the loading velocity and the maximum damping force is not found. Thus, nonlinear modeling of the developed MSDs is essential.

### 2.3. Modeling of the MSDs

To evaluate the seismic performance of the developed MSDs, it is necessary to build a mathematical model which properly represents the relationship between loading velocity and damping force. Since the MSDs do not behave linearly as shown in Table 1, a force-velocity model which can describe the nonlinear properties of the MSDs is adopted as shown in the following equation [24]:

(1) $$\begin{array}{c} F=C_{d}v^{\alpha }, \end{array}$$

where *F* is the damping force of the MSD, *C*_*d*_ is the damping coefficient, *v* is the velocity of the MSD, and *α* is the exponential parameter.

To determine the damping coefficient *C*_*d*_ and the parameter *α*, a nonlinear curve fitting technique [25] is used and the results are summarized in Table 2. The damping coefficients of TSD or PSD are not exactly three times or five times larger than those of SSD, where these losses may be caused by the friction between linkage units. However, the losses are not large and are quite acceptable. The damping coefficient is increased approximately in proportion to the number of slim dampers.

**Figure 5:**
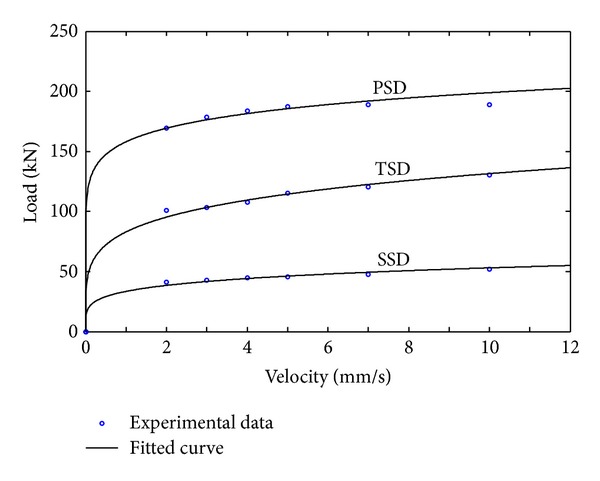
Load-velocity relationship of MSDs.

Figure 5 shows the results of a curve fitting. Not only are these curves well fitted to the observed data but also the nonlinear properties of the SSD, TSD, and PSD are also described properly. Thus, (1) and the values of Table 2 can be considered as an appropriate model for the developed MSDs. These will be used in the numerical simulation and performance evaluation of earthquake excited structures with MSDs in the next section.

## 3. Numerical Simulation

A numerical simulation of an earthquake excited ten-story building is performed to evaluate the seismic performance of the MSDs. In the nonlinear time history analysis, a structural analysis program, MIDAS Gen [26], is used and several historical earthquake records are selected as input excitations. Performances of the MSDs are evaluated according to installation configurations and the total number of MSDs and comparative results are then presented.

### 3.1. Example Structure and Input Excitation

**Figure 6:**
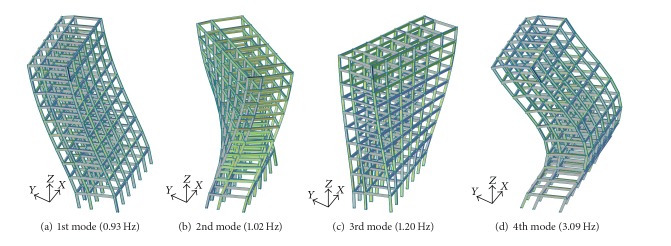
Mode shapes of the example building.

A ten-story steel frame building is selected as an example structure. It is a commercial office building with a width of 9.5 meters and a height of 35 meters. In the modeling of the building, a 3-dimensional frame element is used and a Rayleigh damping of 2% is assumed. The natural frequencies of the first four modes are 0.93 Hz, 1.02 Hz, 1.20 Hz, and 3.09 Hz, respectively. The mode shapes are depicted in Figure 6. Three historical earthquakes with different magnitudes and frequency contents (i.e., El Centro earthquake (1940, PGA 0.35 g), Hachinohe earthquake (1968, PGA 0.24 g), and Taft earthquake (1952, PGA 0.16 g)) are selected as input excitations for nonlinear time history analyses of the building. For the application of the MSD system, a Maxwell model, which is one of the nonlinear connection elements in the MIDAS Gen Program, is adopted. The *C*_*d*_ in Table 2 is used for the damping coefficient of the Maxwell model. For the stiffness parameter in the model, an infinity value is assumed. Also, the boundary nonlinear time history analysis method in the MIDAS Gen Program is used for the dynamic simulation of the building with MSD systems.

### 3.2. Analysis Cases

**Figure 7:**
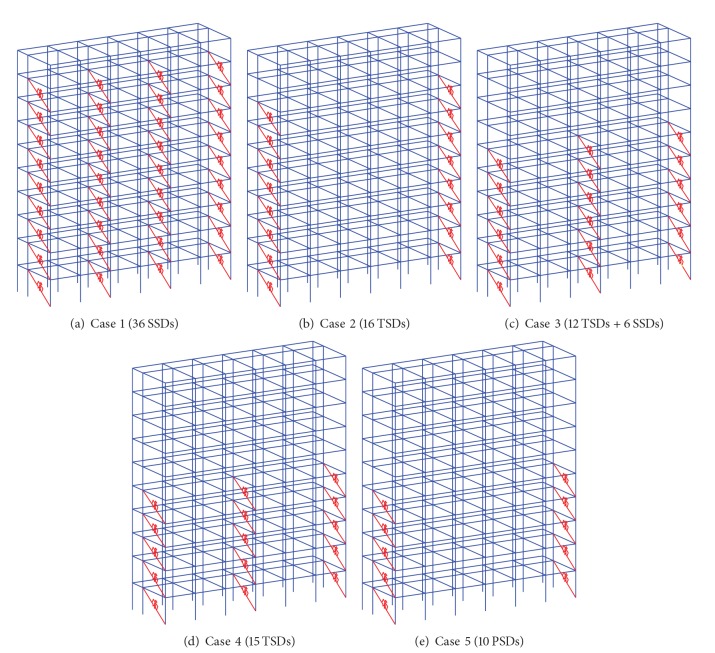
Various locations and combinations of the MSD system.

The seismic performance and effectiveness of the damper system could be mainly affected by the distributed locations and damping capacities of the MSDs [27–30]. To investigate the effectiveness of the MSD system, we performed numerical simulations of 30 cases with various configurations of MSD systems and selected 5 cases that have different damping capacities and installation locations but show similar seismic responses of the building. These are summarized in Table 3 and the installation configurations are depicted in Figure 7. Also, buildings without an MSD system are simulated using three earthquakes.

### 3.3. Earthquake Response Comparison

**Figure 8:**
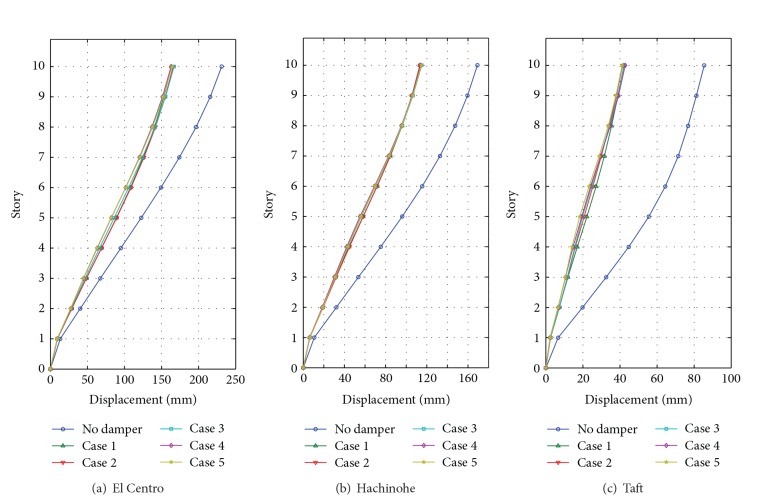
Comparative maximum story displacements subject to input earthquakes.

**Figure 9:**
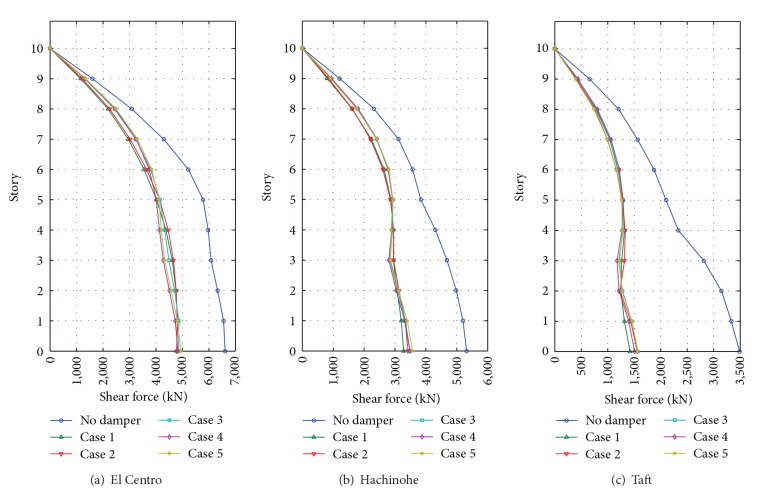
Comparative base shear forces subject to input earthquakes.

As the number of floors increases, the maximum displacement and the base shear force increase due to the self-weight. Thus, we need to investigate the maximum displacement and base shear force in the comparison of earthquake responses. Figures 8 and 9 show the maximum displacement of floors and the maximum base shear, respectively.

In the case of the El Centro earthquake, the maximum top displacement is 231.1 mm for the building without a damper, whereas the maximum displacements for the building with MSDs are 163.4 mm (Case  1), 166.8 mm (Case  2), 166.8 mm (Case  3), 162.8 mm (Case  4), and 164.1 mm (Case  5). That is, the decrease ratios of responses are about 27%–30% for all cases. The maximum base shear is 6,611 kN for the structure without a damper. The maximum base shear of structures with MSD is 4,815 kN (Case  1), 4,808 kN (Case  2), 4,863 kN (Case  3), 4829 kN (Case  4), and 4,940 kN (Case  1). The reduction ratios are about 25%–28%.

For the Hachinohe earthquake responses, the maximum top displacement is 169.2 mm for the structure without a damper, whereas those of structures with MSDs are 113.4 mm (Case  1), 113.4 mm (Case  2), 115.1 mm (Case  3), 114.4 mm (Case  4), and 114.7 mm (Case  5), which corresponds to 32%-33% decreased responses. The maximum base shear of a structure with MSD is decreased by about 33%–38% compared with the original response. In the case of the Taft earthquake, the maximum top displacement is 85.5 mm for the structure without a damper, and that of structures with MSDs is 42.1 mm (Case  1), 41.3 mm (Case  2), 42.3 mm (Case  3), 42.8 mm (Case  4), and 41.2 mm (Case  5), which are about 50% of the original response. The maximum base shear is 3488 kN for the structure without a damper, whereas those of structures with MSDs are 1421 kN (Case  1), 1558 kN (Case  1), 1570 kN (Case  1), 1504 kN (Case  1), and 1568 kN (Case  1), which are about a 55%–60% decrease of the base shear force.

From the comparative results above, it is found that the proposed MSD system can effectively reduce the seismic responses. In addition, it is observed that the five simulated cases show similar seismic performances though they have different installation configurations and total capacities.

However, it should be noted that the results of seismic performance of the building with MSD system are limited to numerical simulation. For further verification and dynamic tests are required.

## 4. Evaluation of Cost-Effectiveness

Simulated results show that seismic performances can be improved by appropriate combinations or distributions of the MSD system. The five simulated cases show a similar level of responses but have different capacities and configurations of the MSD system. This implies that a more effective system can be obtained, which requires less damping capacity but exhibits desirable seismic performance. Therefore, it is important to investigate the cost-effectiveness of the MSD system according to various installation configurations.

**Figure 10:**
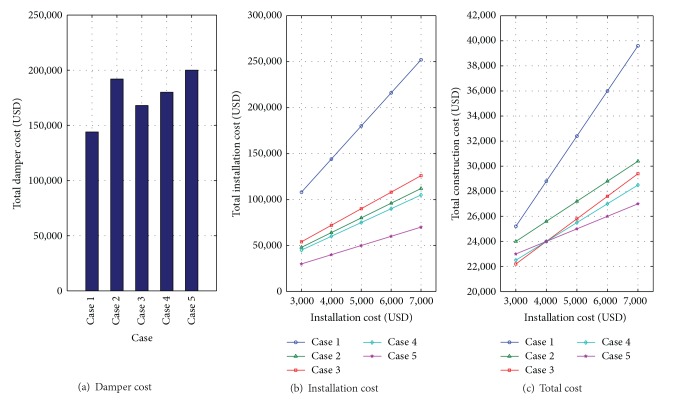
Cost of MSD systems (Case  1~Case  5).

To evaluate the cost-effectiveness of MSDs, five cases (Case  1 to Case  5) which show similar seismic performances are compared. A ready-made single slim damper (SSD) is assumed to cost 4000 USD, which is the average price of a 30 kN capacity viscous damper. Therefore, the price of dampers used for each case is set to be fixed as shown in Figure 10(a). However, the installation costs of MSDs are assumed to vary from 3000 USD to 7000 USD, considering the difficulty level of various installation cases. The installation costs are presented in Figure 10(b). Finally, the total cost is defined as the sum of the damper cost and installation cost, and the result is shown in Figure 10(c).

The cost of dampers is the lowest in Case 1, where the number of SSDs is the smallest (36 units), and is the highest in Case 5, where the number of SSDs is the largest (50 units). On the contrary, the increasing rate of the installation cost is the highest for Case 1, where 36 installations are necessary. Case 2, Case 3, and Case  4 have similar installation costs. For Case 5, the rate of increase of installation cost is the lowest, as presented in Figure 10(b).

For Case 1, the damper cost is the smallest, but a total of 36 places (4 places per floor) are required for the installation of dampers, and the total cost is not the most efficient. Additionally, two inner installations of dampers can cause limitations on space serviceability. For Case 2, only two dampers per floor are installed at both sides and it is efficient in the sense of the serviceability of the space. However, the use of many SSDs increases the installation cost and it is not a cost-efficient case. For Case 3 and Case 4, one inner installation of the damper can cause slight limitation on space serviceability, but it can be considered as the most cost-efficient case if the installation cost per unit is less than 4000 USD. For Case 5, dampers are installed at both ends of each floor. The space variability and serviceability are relatively better than in other cases. In addition, if the installation cost per one place is over 4000 USD, it is the most efficient method.

The evaluated results can be concluded as follows. When the installation cost per space is less than 4000 USD, Case 3 or Case 4 is an efficient system for the building and Case 5 is more efficient than other cases when the cost is over 4000 USD because the number of installations is the smallest.

## 5. Conclusions 

The evaluation of seismic performance and cost-effectiveness of the multiple slim-type damper system is performed in this study. A new MSD system is developed for the vibration control of earthquake excited buildings. The developed system requires smaller installation spaces than conventional damping devices, whereas it can maintain acceptable seismic performances. Three MSDs, that is, SSD, TSD, and PSD, are manufactured at a real scale and loading tests are carried out. From the cyclic loading tests, reliable energy dissipation behavior is clearly observed. To validate the practical applicability, nonlinear time history analysis is performed for a ten-story earthquake excited building structure. The seismic performance and cost-effectiveness of the MSD system are also investigated according to the various installation configurations of the MSD system. From the comparative results of numerical simulations and cost-effectiveness evaluation, it is shown that the MSD system is quite feasible and the combination of MSD systems can effectively improve the seismic performance of earthquake excited building structures.

## Figures and Tables

**Table 1:** Maximum damping forces of the MSDs.

Velocity (mm/sec)	SSD max. force (kN)	TSD max. force (kN)	PSD max. force (kN)
2	41.20	100.99	169.42
3	42.77	103.30	178.39
4	44.78	107.62	183.55
5	45.76	115.07	187.22
7	47.58	120.52	189.04
10	51.89	130.33	188.99

**Table 2:** Damping coefficient and exponential parameter of the MSDs.

Type	SSD	TSD	PSD
Damping coefficient *C*_*d*_ (kN sec/mm)	33.5	83.0	133.5
Exponential parameter α	0.2	0.2	0.2

**Table 3:** Five cases for numerical simulation.

	Installation configuration
Case 1	4 SSDs at 1st~9th floor (36 SSDs)
Case 2	2 TSDs at 1st~8th floor (16 TSDs)
Case 3	2 TSDs at 1st~6th floor (left and right sides) and 1 TSD at 1st~6th floor (center) (12 TSDs + 6 SSDs)
Case 4	3 TSDs at 1st~5th floor (15 TSDs)
Case 5	2 PSDs at 1st~5th floor (10 PSDs)
